# Effects of lornoxicam on the physiology of severe sepsis

**DOI:** 10.1186/cc2969

**Published:** 2004-10-27

**Authors:** Dilek Memiş, Beyhan Karamanlıoğlu, Alparslan Turan, Onur Koyuncu, Zafer Pamukçu

**Affiliations:** 1 Associate Professor, Department of Anaesthesiology and Reanimation, Medical Faculty, Trakya University, Edirne, Turkey; 2 Professor, Department of Anaesthesiology and Reanimation, Medical Faculty, Trakya University, Edirne, Turkey

**Keywords:** biochemical parameters, cytokine levels, haemodynamic parameters, intensive care unit, lornoxicam, outcome, severe sepsis

## Abstract

**Introduction:**

The purpose of the present study was to evaluate the effects of intravenous lornoxicam on haemodynamic and biochemical parameters, serum cytokine levels and patient outcomes in severe sepsis.

**Methods:**

A total of 40 patients with severe sepsis were included, and were randomly assigned (20 per group) to receive either lornoxicam (8 mg administered intravenously every 12 hours for six doses) or placebo. For both groups the following were recorded: haemodynamic parameters (heart rate, mean arterial pressure), nasopharyngeal body temperature, arterial blood gas changes (pH, partial oxygen tension, partial carbon dioxide tension), plasma cytokine levels (IL-1β, IL-2 receptor, IL-6, IL-8, tumour necrosis factor-α), biochemical parameters (lactate, leucocytes, trombocytes, creatinine, total bilirubin, serum glutamate oxalate transaminase), length of stay in the intensive care unit, duration of mechanical ventilation and mortality. All measurements were obtained at baseline (before the start of the study) and at 24, 48 and 72 hours from the start of lornoxicam/placebo administration.

**Results:**

No significant differences were found between the intravenous lornoxicam and placebo groups in major cytokines, duration of ventilation and length of intensive care unit stay, and inspired fractional oxygen/arterial oxygen tension ratio (*P *> 0.05).

**Conclusion:**

In these patients with severe sepsis, we found intravenous lornoxicam to exert no effect on haemodynamic and biochemical parameters, cytokine levels, or patient outcomes. Because of the small number of patients included in the study and the short period of observation, these findings require confirmation by larger clinical trials of intravenous lornoxicam, administered in a dose titrated manner.

## Introduction

Sepsis is defined as the systemic response to infection [1,2]. The deleterious effects of bacterial invasion of body tissues results from the combined actions of enzymes and toxins produced by the micro-organisms themselves, and the actions of endogenous cells in response to the infectious process. Despite advances in supportive care, mortality rates in patients with severe sepsis continue to exceed 30%. During sepsis vasoactive arachidonic acid metabolites of the cyclo-oxygenase (COX) pathway are released. In particular, thromboxane A_2 _and prostacyclin have been found to be elevated in sepsis [3,4]. Thromboxane A_2 _has been associated with bronchoconstriction, vasocontriction and platelet aggregation [3]. Prostacyclin, the predominant eicosanoid generated by activated endothelial cells, is a powerful vasodilator and antagonist of thrombosis [3]. Prostaglandin (PG)E_2 _is among the most potent and inducible of the prostanoids that are produced in states of inflammation. Specifically, there is evidence to support roles for PGE_2 _as a mediator of sepsis-induced immunosuppression, an inhibitor of proinflammatory cytokine expression from monocytes, and an inducer of IL-10 production [5-7]. Conversely, PGE_2 _has been shown to mediate detrimental effects in sepsis, including vasodilation and increased vascular permeability [8]. In addition, its role as a mediator in fever induction and augmentation of pain is well established [9]. Several studies [10-12] conducted in endotoxin-challenged animals have found beneficial effects of nonselective COX inhibitors. These beneficial effects were felt to be mediated, in part, by mitigation of pathophysiological events in sepsis induced by PGs.

COX exists as two isoforms – COX-1 and COX-2. The former is constitutively expressed, whereas COX-2 is expressed at low levels in most normal resting cells. Marked upregulation of COX-2 occurs in synoviocytes, macrophages and endothelial cells during stress and in inflammatory conditions such as sepsis. COX-2 expression is induced by a number of cytokines, including tumour necrosis factor (TNF) and IL-1, mitogens and growth factors, lipopolysaccharide (LPS), and other inflammatory stimuli [13]. Recent studies [14,15] provided evidence suggesting that selective COX-2 inhibitors have significant advantages over their nonselective counterparts. The specific benefits of COX-2 inhibitors include decreased gastrointestinal toxicity and bleeding [14,16].

As with other nonsteroidal anti-inflammatory drugs (NSAIDs), lornoxicam inhibits PG synthesis via inhibition of COX, but it does not inhibit 5-lipoxygenase. The ratio of inhibitory potency of human COX-1 to COX-2 for lornoxicam is 0.6 [17]. Lornoxicam was reported to be 100-fold more potent than tenoxicam in inhibiting PGD_2 _formation in rat polymorphonuclear leucocytes *in vitro*, and it was more active than indomethacin and piroxicam in preventing arachidonic acid induced lethality in mice *in vivo *[17]. Lornoxicam also inhibited the formation of nitric oxide in RAW264.7 mouse macrophages stimulated with endotoxin, indicating an effect on inducible nitric oxide synthase [18]. It also exhibited marked inhibitory properties on endotoxin-induced IL-6 formation in THP1 monocytes, with less activity on TNF and IL-1β. It appears that lornoxicam, in addition to markedly inhibiting COX and inducible nitric oxide synthase, has a moderate effect on the formation of proinflammatory cytokines [19].

The purpose of the present study was to evaluate the effects of intravenous lornoxicam on serum cytokine levels, haemodynamic and biochemical parameters, and outcomes in humans with severe sepsis.

## Methods

### Patient population and study design

The regional committee on medical research ethics approved the study. Written informed consent was obtained, directly from the patients wherever possible or from the next of kin. Critically ill patients with bacteriologically documented infections were included in the study as soon as they met at least two of the following criteria for sepsis, as defined by the American College of Chest Physicians/Society of Critical Care Medicine Consensus Conference Committee [2]: temperature >38°C or <36°C; heart rate >90 beats/min; respiratory rate >20 breaths/min or arterial carbon dioxide tension <32 mmHg; and leucocyte count >12 × 10^9 ^cells/l or <4 × 10^9 ^cells/l. In addition, at least one of following conditions was required: hypoxaemia (arterial oxygen tension/fractional inspired oxygen ratio <250); oliguria (urine output <0.5 ml/kg body weight for 2 hours); lactic acidosis (lactate concentration >2 mmol/l); thrombocytopaenia (platelet count <100 × 10^9^/l); and a recent change in mental status without sedation. Patients who were younger than 18 years, had known or suspected hypersensitivity to COX inhibitors, or had received a COX inhibitor within 12 hours (or aspirin within 24 hours) were enrolled in another experimental protocol (not part of the present study), or were excluded if consent could not be obtained. Also excluded were patients with known or suspected brain death; those with advanced acute or chronic renal or hepatic failure; those who had received potent immunosuppressive drugs; those with gastrointestinal bleeding; those who were pregnant; and those with a known irreversible underlying disease, such as end-stage neoplasm.

The Acute Physiology and Chronic Health Evaluation (APACHE) II score [20] and Sepsis-related (or Sequential) Organ Failure Assessment (SOFA) score [21] (Table 1) were employed to determine the initial severity of illness.

If required, patients underwent surgical procedures before the start of the study. No invasive surgery was performed during the 72-hour study period. All patients were ventilated in volume-controlled mode (Puritan Bennett 7200; Carlsbad, CA) and received continuous analgesic sedation with midazolam and fentanyl. Ventilator settings, level of positive end-expiratory pressure and fractional inspired oxygen were kept constant during intravenous administration of lornoxicam or placebo. Antibiotic treatment was adjusted according to the results of bacteriological culture, such as blood culture or culture of samples taken from different body sites. In all participants fluid replacement was administered to maintain central venous pressure between 4 and 8 mmHg. No inotropic agent was administered during the study. Those patients who met the criteria for severe sepsis presented above were enrolled in the study within 8 hours of intensive care unit (ICU) admission.

### Protocol

Randomization was done using a computer-steered permuted block design. The study was planned prospective, randomized, double blind, and placebo controlled. In order to perform the study in a double-blind manner, drug solution was administered to all patients by a nurse who had no knowledge of the study protocol, and follow up was done by an anaesthetist who also had no knowledge of the study protocol. Twenty patients received lornoxicam 8 mg (Xefo; Abdi Ýbrahim, Istanbul, Turkey), administered intravenously every 12 hours for a total of six doses. In the placebo group, also including 20 patients, saline was administered using the same volume and dosing regimen.

### Measurements

All patients had arterial catheters placed (Abbott Transpac^® ^IV; Abbott, Sligo, Ireland) and central venous catheters placed via subclavian (Certofix trio V 720 7F×8"; Braun, Melsungen, Germany). Arterial blood samples were simultaneously withdrawn for measurements of pH, partial oxygen tension, partial carbon dioxide tension and arterial oxygen saturation (Medica Easy BloodGas; Massachusetts, USA). Central venous pressure, mean arterial pressure, heart rate and naso-opharyngeal temperature were continuously monitored (Space Labs Inc., Redmond, WA, USA). All measurements were obtained at baseline (before the start of the study) and again at 24, 48 and 72 hour after the start of infusion. Lactate, platelets, leucocytes, bilirubin, alanine aminotransferase and creatinine were determined at the same times (Vitalab Flexor, Dieren, The Netherlands).

TNF-α, IL-1β, IL-2 receptor, IL-6 and IL-8 levels were measured at the same times. Venous blood was collected into a 10 ml sterile plain tube (without anticoagulant) before administration of any medications and stored at -20°C. Before assay, all samples were thawed to room temperature and mixed by gentle swirling or inversion. All sera were assayed on the same day to avoid interassay variation. TNF-α, IL-1, IL-2 receptor, IL-6 and IL-8 levels were measured using a solid-phase, two-site chemiluminescent enzyme immunometric assay method (Immulite TNF-α, Immulite IL-1β, Immulite IL-2 receptor, IL-6 Immulite and IL-8 Immulite; EURO/DPC, Llanberis, UK). The antibodies used in this procedure have no known cross-reactivities with other cytokines. The intra-assay and interassay coefficients of variation, respectively, for this procedure were as follows: for IL-1β, 2.8–4.9% and 4.8–9.1%; for IL-2 receptor, 2.9–3.7% and 6.1–8.1%; for IL-6, 3.6–6.2% and 5.4–9.6%; for IL-8, 3.6–3.8% and 5.2–7.4%; and for TNF-α, 2.6–3.6% and 4.0–6.5%. The lowest detectable limits of IL-1β, IL-2 receptor, IL-6, IL-8 and TNF-α were 1.5 pg/ml, 5 U/ml, 5 pg/ml, 2 pg/ml and 1.7 pg/ml, respectively.

The duration of mechanical ventilation was recorded. Survival was defined as being alive at hospital discharge.

### Statistical analysis

Repeated measures analysis of variance was used to evaluate the differences between and within groups from baseline. In the case of statistical significance, groups were tested by independent sample t-test to determine which difference was significant. Data are expressed as mean ± standard deviation. *P *< 0.05 was considered statistically significant.

## Results

### Patient characteristics

Clinical and demographic characteristics of the patients are listed in Table 2. Of the 40 patients included, 20 received intravenous lornoxicam and 20 received placebo. Fifteen patients had septic shock on admission (seven [35%] in the lornoxicam group and eight [40%] in the placebo group) and died while in the ICU. Baseline APACHE II scores (17.10 ± 3.58 and 18 ± 3.72 in the lornoxicam and placebo groups, respectively) and SOFA scores (5.90 ± 1.72 and 6.20 ± 2.2) were similar in the two groups (*P *> 0.05). SOFA scores at 24 hours (5.50 ± 1.52 and 6.1 ± 1.2 in the lornoxicam and placebo groups, respectively), 48 hours (5.60 ± 1.6 and 6.0 ± 1.3) and 72 hours (5.72 ± 1.4 and 6.1 ± 1.6) were also similar (*P *> 0.05). Infection was documented in all patients.

### Haemodynamic parameters and oxygen transport variables

There were no significant differences between groups with respect to pH, partial oxygen tension, partial carbon dioxide tension, arterial oxygen tension/inspired fractional oxygen ratio and arterial oxygen saturation (*P *> 0.05). No significant changes in mean arterial pressure and heart rate were found in either group (Table 3). There were no significant differences between groups in biochemical parameters (Table 4; *P *> 0.05).

### Outcomes

Outcomes are listed in Table 2. In the ICU, the overall mortality rates were 35% (seven patients out of 20) in the lornoxicam group and 40% (eight patients out of 20) in the placebo group (*P *> 0.05). All of those who died did so while they were being mechanically ventilated. In the lornoxicam and placebo groups the mean durations of ventilation were 6.1 ± 2.4 and 5.8 ± 3.1 days, respectively (*P *> 0.05). The length of ICU stay in lornoxicam treated survivors was not significantly different from that of placebo treated survivors (10.2 ± 7.1 versus 9.2 ± 8.4 days; *P *> 0.05).

### Plasma cytokine levels

TNF-α, IL-1β, IL-2 receptor, IL-6 and IL-8 levels remained unchanged during the study (Table 5).

### Side effects

Intravenous lornoxicam was well tolerated by all patients, and no side effects were noted during or after administration of lornoxicam.

## Discussion

Systemic inflammatory response leading to postoperative organ dysfunction and sepsis remains a formidable clinical challenge and carries a significant risk for mortality. Sepsis and septic shock remain major causes of death in ICUs. A number of studies have examined the role of nonselective COX inhibitors both in animal models of sepsis and in patients with sepsis syndrome. Several studies [10-12] demonstrated beneficial effects of nonselective COX inhibition, predominantly in endotoxin-treated animals. However, subsequent studies [22,23] examining the role played by NSAIDs, particularly ibuprofen, in human sepsis trials have been disappointing. The present study was therefore conducted to determine whether COX inhibition is upregulated early after the onset of severe sepsis, and if so whether COX inhibition prevents the occurrence of septic shock.

The arachidonic acid pathway is highly activated in macrophages, monocytes and other inflammatory cells, resulting in the formation of eicosonoids. PGs are involved in all phases of the inflammatory process, including fever and pain reactions, as well as in a large number of physiological functions, including intestinal motility, platelet aggregation, vascular tone, renal function and gastric secretion, among others. Two COX isoforms have been identified: COX-1 and COX-2. The former is a constitutive enzyme that is expressed in many cells as a house-keeping enzyme and stimulates homeostatic production of PGs. COX-2 is an inducible form of the enzyme that is expressed at the onset of inflammation by many cell types that are involved in the inflammatory response. NSAIDs act mainly through COX inhibitors, thus preventing the formation of proinflammatory prostanoids. Lornoxicam, a new member of the oxicam class of NSAIDs, inhibits PG synthesis via inhibition of COX, but it does not inhibit 5-lipoxygenase. Lornoxicam is at least 10 times more potent as an anti-inflammatory agent than piroxicam, and 12 times more potent as an analgesic than tenoxicam [17,19].

The primary pharmacological action of NSAIDs is, of course, to decrease the formation of PGs and thromboxanes by inhibiting COX, a key enzyme in the biochemical pathway that leads to formation of these potent mediators [24]. Accordingly, products of the COX pathway, sometimes referred to as 'prostanoids', have been implicated in the pathogenesis of the deleterious systemic consequences of serious infection and/or endotoxaemia. In addition, the toxic effects of TNF (thought to be one of the primary cytokines responsible for LPS-induced lethality) can be ameliorated by treating mice or rats with NSAIDs such as indomethacin or ibuprofen [25]. NSAIDs have been shown to increase release cytokines (TNF, IL-6, or IL-8) by stimulated mononuclear cells *in vitro *[26,27].

Complications of sepsis have been related to an intense host response based on a delicate equilibrium between various proinflammatory and anti-inflammatory mediators [28]. Overwhelming production of proinflammatory cytokines, such as TNF-α, IL-1β, IL-2 receptor, IL-6 and IL-8, may induce biochemical and cellular alterations either directly or by orchestrating secondary inflammatory pathways.

Reddyl and coworkers [5] evaluated the effect of pretreatment with NS-398, a highly selective COX-2 inhibitor, on survival and inflammatory mediator production in two models of sepsis in mice (LPS challenge and peritonitis induced by caecal ligation and puncture [CLP]). They found that selective inhibition of COX-2 resulted in improvement in early survival in murine endotoxaemia but not in a more physiologically relevant model of abdominal sepsis (CLP). The early improvement in survival in endotoxin-challenged animals was not attributable to changes in inflammatory cytokine expression or organ-specific neutrophil sequestration. Pretreatment with NS-398 failed to improve long-term survival in either of the models studied, although in the endotoxaemia model administration of the COX-2 inhibitor had a modest salutary effect on early mortality. In addition, although treatment with NS-398 blocked LPS-induced increases in the circulating levels of immunoreactive PGE_2_, injection of the COX-2 inhibitor did not modulate plasma concentrations of TNF or the CXC chemokine KC.

Knoferl and coworkers [29] also evaluated the effect of pretreatment with NS-398, that trauma/haemorrhage results in activation of Kupffer cells to release inflammatory mediators and it leads to immunosuppression. *In vitro *production of IL-6 by Kupffer cells after CLP was significantly reduced by *in vivo *NS-398 treatment. However, NS-398 had no effect on TNF-α levels *in vivo *or *in vitro*. Strong and coworkers [12] showed that administration of NS-398 for 24 hours after trauma improved survival when mice were subjected to CLP and puncture 7 days later. It is noteworthy that NS-398 exhibited protective effects in two models of sepsis characterized by infection in the setting of trauma-induced immunosuppression, whereas the drug was largely ineffective when sepsis was induced in immunocompetent animals. Dallal and coworkers [30] demonstrated that T-cell suppression during neonatal sepsis is accompanied by a decrease in IL-2 production. Such suppression was ameliorated by COX-2 inhibitor, suggesting a role for PGE_2 _in suppressed T-cell-mediated immune function in neonatal sepsis. Arons and colleagues [22] compared the clinical and physiological characteristics of febrile septic patients with those of hypothermic septic patients, and compared plasma levels of cytokines TNF-α and IL-6 and thromboxane B_2 _and prostacyclin between hypothermic septic patients and febrile patients. They administered ibuprofen but found that this drug had no effect on cytokine levels.

Reddyl and coworkers [5] indicated that pharmacological inhibition of COX-2 has only very modest effects on outcome in experimental sepsis or endotoxaemia. Because these findings are discrepant with respect to those obtained with isoform nonselective agents, it is regrettable that those investigators did not include a 'positive control' arm in their studies to evaluate the effects of treatment with an agent such as indomethacin or ibuprofen in their laboratory's models of sepsis. In our study we did not observe any significant changes in systemic cytokine levels during NSAID administration in humans with severe sepsis. Cytokine levels in plasma do not necessarily reflect local synthesis of cytokines by cells. Many cells have surface receptors for these cytokines with high binding properties, and target cells and soluble receptors trap cytokines. Thus, cytokines released at the local level may remain undetected in plasma. In the present study we found plasma cytokine levels to remain unchanged over a period of 72 hours.

Wang and coworkers [31] conducted a study to determine whether inhibition of PGI_2 _synthesis prevents the hyperdynamic response in early sepsis in animals. Those investigators found that inhibition of PGI_2 _production did not prevent the hyperdynamic and hypercardiovascular responses during early sepsis; hence, mediators other than PGI_2 _appear to play a major role in producing the hyperdynamic response under such conditions. Fox and colleagues [32] postulated that the attenuated pulmonary and systemic vascular contractility observed in sepsis was secondary to the release of vasodilator PGs. They used the COX inhibitor meclofenamate to inhibit PG synthesis in a model of hyperdynamic sepsis, and found that meclofenamate had no effect on either the pulmonary or systemic response to phenylephrine infusion in septic animals. However, Wanecek and coworkers [11] demonstrated that endotoxin-induced pulmonary hypertension in the pig can be prevented with a combination of the nonpeptide mixed endothelin receptor antagonist bosentan and the COX inhibitor diclofenac. They found that the combination of bosentan and diclofenac induced systemic and pulmonary vasodilatation. During endotoxin shock, this drug combination efficiently counteracted pulmonary hypertension and improved cardiac performance, and splenic and renal blood flows. These favourable circulatory effects might have resulted in a reduction in both sympathetic nervous system activation and metabolic acidosis. In the present study we found that lornoxicam had no effect on the cardiovascular and pulmonary systems in severe sepsis in humans, but our study was designed to assess the effects of lornoxicam treatment given before septic shock but after systemic inflammatory response syndrome. For this reason we identified no serious cardiovascular and pulmonary system problems in the patients studied.

Arons and coworkers [22] compared clinical and physiological characteristics of febrile septic patients with those in hypothermic septic patients, and compared plasma levels of cytokines TNF-α and IL-6, and thomboxane B_2 _and prostacyclin between hypothermic septic patients and febrile patients. Those investigators found that ibuprofen treatment had a positive impact on vital signs, organ failure and mortality in hypothermic septic patients, and concluded that ibuprofen could substantially decrease mortality in this selected group of septic patients. In our study we found that lornoxicam had no effect on vital signs and mortality in patients with severe sepsis. The overall ICU mortality rate was 37.5% (15 patients out of 40) in total, and these deaths were all attributable to septic shock. However, all of the patients died after completion of the study.

Lornoxicam has been shown to produce less gastric toxicity than its nonselective counterparts. This may be especially important in critically ill patients, who are at significantly greater risk for developing gastric ulceration. In addition, the lack of inhibitory effect on platelet function, which occurs with the use of COX-2 selective compounds, may decrease the incidence of bleeding complications [17,19]. In the present study we did not identify any lornoxicam related adverse effects.

In summary, we found that intravenous lornoxicam had no effect on haemodynamic and biochemical parameters, cytokine levels, or patient outcomes in severe sepsis. Selective inhibition of COX-2 in sepsis requires further study. However, the findings reported here, indicating that lornoxicam lacks benefit in patients with severe sepsis, are disappointing.

## Key messages

• 	Administration of intravenous lornoxicam appeared to confer no benefit in patients with severe sepsis.

## Competing interests

The author(s) declare that they have no competing interests.

## Abbreviations

APACHE = Acute Physiology and Chronic Health Evaluation; CLP = caecal ligation and puncture; COX = cyclo-oxygenase; ICU = intensive care unit; IL = interleukin; LPS = lipopolysaccharide; NSAID = nonsteroidal anti-infllammatory drug; PG = prostaglandin; SOFA = Sepsis-related (Sequential) Organ Failure Assessment; TNF = tumour necrosis factor.

## Figures and Tables

**Table 1:** Sepsis-related (or Sequential) Organ Failure Assessment (SOFA) scores

Parameter	SOFA score				
	
	0	1	2	3	4
Respiration (PaO_2_/FiO_2 _ratio)	>400	≤ 400	≤ 300	≤ 200 with respiratory support	≤ 100
Coagulation (platelets × 10^3^/mm^3^	>150	≤ 150	≤ 100	≤ 50	≤ 20
Liver (bilirubin [mg/dl (μmol/l)])	<1.2 (<20)	1.2–1.9 (20–32)	2.0–5.9 (33–101)	6.0–11.9 (102–204)	>12.0 (>204)
Cardiovascular (hypotension)	No hypotension	MAP <70 mmHg	Dopamine ≤ 5 or dobutamine at any dose	Dopamine>5 or adrenaline (epinephrine) ≤ 0.1 noradrenaline (norepinephrine) ≤ 0.1	Dopamine >15 or adrenaline >0.1 noradrenaline >0.1
Central nervous system (GCS score)	15	19–14	10–12	6–9	<6
Renal (creatine [mg/dl] or urine output)	<1.2	1.2–1.9	2.0–3.4	3.5–4.9 or <500 ml/day	>5 or <200 ml/day

FiO_2_, fractional inspired oxygen; GCS, Glasgow Coma Scale; MAP, mean arterial pressure;

PaO_2_, arterial oxygen tension.

**Table 2:** Demographic and clinical characteristics of lornoxicam treated and placebo patients

Characteristic	Lornoxicam group (*n *= 20)	Placebo group (*n *= 20)
Age (years [range])	49 (19–87)	51 (20–89)
Sex (male/female)	13/7	9/11
Source of infection		
Respiratory	15	17
Gastrointestinal	2	1
Blood	2	1
Urinary tract	1	1
APACHE II score^a^	17.10 ± 3.58	18 ± 3.72
SOFA score^a^	5.90 ± 1.72	6.20 ± 2.2
Duration of ventilation^a^	6.1 ± 2.4	5.8 ± 3.1
Length of stay^a^	10.2 ± 7.1	9.2 ± 8.4
Mortality rate (%)	35	40

There were no significant differences between the groups. ^a^Values are expressed as mean ± standard deviation. APACHE, Acute Physiology and Chronic Health Evaluation; SOFA, Sepsis-related (or Sequential) Organ Failure Assessment.

**Table 3:** Haemodynamic, oxygen and temperature variables

Parameter	Baseline	Hours after the start of infusion		
		
		24	48	72
Heart rate (beats/min)				
Lornoxicam	98 ± 24	99 ± 24	99 ± 22	96 ± 22
Placebo	95 ± 17	100 ± 23	98 ± 23	97 ± 22
Mean arterial pressure (mmHg)				
Lornoxicam	91 ± 16	92 ± 13	89 ± 13	93 ± 13
Placebo	91 ± 18	93 ± 14	91 ± 12	89 ± 11
Arterial pH				
Lornoxicam	7.34 ± 0.08	7.36 ± 0.07	7.33 ± 0.10	7.36 ± 0.06
Placebo	7.35 ± 0.07	7.37 ± 0.07	7.34 ± 0.07	7.37 ± 0.05
PaCO_2 _(torr)				
Lornoxicam	34.8 ± 12.1	34.6 ± 11.8	35.00 ± 9.2	36.54 ± 11.1
Placebo	32.6 ± 10	33.5 ± 10.2	36.3 ± 10.13	34.33 ± 12
PaO_2_/FiO_2 _ratio (torr)				
Lornoxicam	182 ± 68	186 ± 56	188 ± 64	189 ± 76
Placebo	184 ± 76	187 ± 45	181 ± 68	185 ± 68
SaO_2 _(%)				
Lornoxicam	96.1 ± 3	96.1 ± 3.1	95.9 ± 3.8	95.9 ± 4.2
Placebo	96.9 ± 3.1	96.0 ± 3.2	96.0 ± 2.8	95.8 ± 3.9
Temperature (°C)				
Lornoxicam	37.8 ± 0.75	37.2 ± 0.6	37.8 ± 0.5	37.9 ± 0.4
Placebo	37.6 ± 0.57	37.8 ± 0.4	37.6 ± 0.6	37.8 ± 0.5

No significant differences were found between groups. Data are expressed as mean ± standard deviation. FiO_2_, fractional oxygen tension; PaCO_2_, arterial carbon dioxide tension; PaO_2_, arterial oxygen tension; SaO_2_, arterial oxygen saturation.

**Table 4:** Biochemical parameters

Parameter	Baseline	Hours after the start of infusion		
		
		24	48	72
Lactate (mg/dl)				
Lornoxicam	25.2 ± 4.1	25.0 ± 3.9	25.9 ± 5.2	26.2 ± 3.7
Placebo	26.3 ± 3.8	26.7 ± 2.9	27.0 ± 3.1	26.9 ± 4.8
Platelets (×10^9^/l)				
Lornoxicam	192.9 ± 16.5	193.5 ± 15.0	178.1 ± 15.4	182.9 ± 15.6
Placebo	188.5 ± 14.4	190.8 ± 15.1	188.7 ± 13.3	190.5 ± 16.7
Leucocytes (×10^9^/l)				
Lornoxicam	14 ± 8.3	15.8 ± 8.3	16.0 ± 7.8	15.9 ± 6.2
Placebo	13.5 ± 6.6	14.7 ± 4.7	15.7 ± 6.7	14.8 ± 7.4
Bilirubin (mg/dl)				
Lornoxicam	0.89 ± 0.38	0.90 ± 0.45	0.93 ± 0.33	0.90 ± 0.34
Placebo	0.90 ± 0.62	0.91 ± 0.38	0.92 ± 0.28	0.91 ± 0.36
Alanine aminotransferase (IU/l)				
Lornoxicam	35.4 ± 5.5	36.0 ± 10.8	35.1 ± 8.6	36.7 ± 8.4
Placebo	35.4 ± 7.4	35.5 ± 5.2	36.2 ± 6.0	37.4 ± 4.9
Creatinine (mg/dl)				
Lornoxicam	1.13 ± 0.96	1.15 ± 0.85	1.2 ± 0.3	1.28 ± 0.8
Placebo	1.01 ± 0.91	1.1 ± 0.65	1.08 ± 0.7	1.1 ± 0.8

No significant differences were found between groups. Data are expressed as mean ± standard deviation.

**Table 5:** Cytokine levels

Cytokine	Baseline	Hours after the start of infusion		
		
		24	48	72
TNF-α (pg/ml)				
Lornoxicam	25.7 ± 15	27.4 ± 16	26.68 ± 12.9	28.1 ± 16.8
Placebo	24.6 ± 18	25.4 ± 16.9	25.55 ± 11.8	26.3 ± 14.2
Il-1β (pg/ml)				
Lornoxicam	6.4 ± 3.8	6.48 ± 6.07	6.21 ± 3.26	6.57 ± 1.8
Placebo	6.35 ± 1.4	6.2 ± 4.30	6.34 ± 4.2	6.19 ± 2.7
IL-2 receptor (U/ml)				
Lornoxicam	1950 ± 1266	1890 ± 1150	1929 ± 1027	2050 ± 1100
Placebo	2270 ± 1110	2179 ± 1005	2300 ± 1190	2268 ± 1000
IL-6 (pg/ml)				
Lornoxicam	100.6 ± 58	105.2 ± 54	108.5 ± 47	104.8 ± 38
Placebo	114.5 ± 385	115.6 ± 48	113.6 ± 51	116 ± 28
IL-8 (pg/ml)				
Lornoxicam	171.50 ± 35	171.6 ± 19.3	172.1 ± 12.6	168.9 ± 11.3
Placebo	169.55 ± 27	168.3 ± 18.4	169.8 ± 18.2	171.0 ± 18.2

No significant differences were found between groups. Data are expressed as mean ± standard deviation. IL, interleukin; TNF, tumour necrosis factor.
